# Venous thromboembolism (VTE) prevention and diagnosis in COVID-19: Practice patterns and outcomes at 33 hospitals

**DOI:** 10.1371/journal.pone.0266944

**Published:** 2022-05-05

**Authors:** Anna L. Parks, Andrew D. Auerbach, Jeffrey L. Schnipper, Amanda Bertram, Sun Y. Jeon, Bridget Boyle, Margaret C. Fang, Shrirang M. Gadrey, Zishan K. Siddiqui, Daniel J. Brotman, the Hospital Medicine Reengineering Network (HOMERuN)

**Affiliations:** 1 Division of Hematology and Hematologic Malignancies, Department of Medicine, University of Utah, Salt Lake City, UT, United States of America; 2 Division of Hospital Medicine, Department of Medicine, University of California, San Francisco, San Francisco, CA, United States of America; 3 Division of General Internal Medicine, Brigham and Women’s Hospital, Boston, MA, United States of America; 4 Division of Hospital Medicine, Department of Medicine, Johns Hopkins School of Medicine, Baltimore, MD, United States of America; 5 Division of Geriatrics, University of California, San Francisco and San Francisco VA Medical Center, San Francisco, CA, United States of America; 6 Division of General, Geriatric, Palliative and Hospital Medicine, University of Virginia, Charlottesville, VA, United States of America; Università degli Studi di Milano, ITALY

## Abstract

**Background:**

Early reports of increased thrombosis risk with SARS-CoV-2 infection led to changes in venous thromboembolism (VTE) management. Real-world data on the prevalence, efficacy and harms of these changes informs best practices.

**Objective:**

Define practice patterns and clinical outcomes related to VTE diagnosis, prevention, and management in hospitalized patients with coronavirus disease-19 (COVID-19) using a multi-hospital US sample.

**Methods:**

In this retrospective cross-sectional study of 1121 patients admitted to 33 hospitals, exposure was dose of anticoagulant prescribed for VTE prophylaxis (standard, intensified, therapeutic), and primary outcome was VTE (pulmonary embolism [PE] and deep vein thrombosis [DVT]); secondary outcomes were PE, DVT, arterial thromboembolism (ATE), and bleeding events. Multivariable logistic regression models accounting for clustering by site and adjusted for risk factors were used to estimate odds ratios (ORs). Inverse probability weighting was used to account for confounding by indication.

**Results:**

1121 patients (mean age 60 ± 18, 47% female) admitted with COVID-19 between February 2, 2020 and December 31, 2020 to 33 US hospitals were included. Pharmacologic VTE prophylaxis was prescribed in 86%. Forty-seven patients (4.2%) had PE, 51 (4.6%) had DVT, and 23 (2.1%) had ATE. Forty-six patients (4.1%) had major bleeding and 46 (4.1%) had clinically relevant non-major bleeding. Compared to standard prophylaxis, adjusted odds of VTE were 0.67 (95% CI 0.21–2.1) with no prophylaxis, 1.0 (95% CI 0.06–17) with intensified, and 3.0 (95% CI 0.89–10) with therapeutic. Adjusted odds of bleeding with no prophylaxis were 5.6 (95% CI 3.0–11) and 5.3 (95% CI 3.0–10) with therapeutic (no events on intensified dosing).

**Conclusions:**

Therapeutic anticoagulation was associated with a 3-fold increased odds of VTE and 5-fold increased odds of bleeding. While higher bleeding rates with high-intensity prophylaxis were likely due to full-dose anticoagulation, we conclude that high thrombosis rates were due to clinical concern for thrombosis before formal diagnosis.

## Introduction

Early in the coronavirus disease-19 (COVID-19) pandemic, reports surfaced suggesting that patients with COVID-19 are at high risk of venous and arterial thrombosis (VTE, ATE) and that standard prophylaxis dosing appeared inadequate [1–5]. These concerns led to some hospital and international society guidelines recommending aggressive VTE prophylaxis strategies in hospitalized patients, particularly in those with elevated D-dimer, including full-dose (therapeutic) anticoagulation in patients without proven VTE [6]. Optimal dosing of anticoagulants and antithrombotic agents in COVID-19 is the subject of randomized clinical trials [7–11]. However, information on the prevalence, efficacy, and potential harms of prophylactic and management strategies that differ from standard guidelines for inpatients offers important insight on real-world management of COVID-related VTE. In addition, concerns about infection control and limited personal protective equipment led to variation in how VTE is diagnosed, such as using point-of-care testing rather than formal radiographic diagnosis [12]; the prevalence of this practice has not been fully described. Thus, the goal of this study was to define current practice patterns and clinical outcomes related to VTE diagnosis, prevention, and management in hospitalized patients with COVID-19 in a multi-hospital US sample.

## Materials and methods

### Study design, participants and data collection

This was a retrospective, multicenter, cross-sectional study. The HOMERuN Network is a Hospitalist collaborative of approximately 70 US academic medical centers (**S1 Table in S1 File**) [13]. Each participating institution identified all patients with a diagnosis of COVID-19 at their institution admitted to general medicine wards or the intensive care unit (ICU); the central coordinating site (Johns Hopkins School of Medicine) randomly selected a sample for chart review. Reviewers were asked to (1) confirm diagnosis of COVID-19 within 30 days based on positive SARS-CoV-2 polymerase chain reaction-based testing, and (2) ascertain whether the hospitalization was attributable to COVID-19. With multiple hospitalizations, only the incident COVID-19 admission was included. Each site was expected to extract data on at least 20 patients but could obtain more, up to a maximum of 100. To obtain accurate exposures and outcomes, we excluded patients admitted for less than 24 hours or transferred to another acute care hospital (**S1 Fig cohort flow diagram in S1 File**).

Reviewers manually extracted data from the electronic health record (EHR), including clinician notes, pharmacy records, laboratory values and radiology reports. Data were uploaded using a standardized electronic data collection tool in REDCap [14]. All available records were reviewed for outcomes up to 30 days post-discharge. For post-discharge events, available outside hospital records were also reviewed. To estimate cumulative inter-rater reliability across sites, we randomly selected two patients from each site duplicate data collection by a second abstractor. Deidentified information was uploaded to a password-protected database. Institutional Review Boards at participating institutions approved the project.

### Measures

We collected information on sociodemographics, medications, radiologic studies, markers of illness severity, length of stay, mortality and comorbid conditions that are included in the Padua score (a widely used risk score that categorizes patients with a score greater than 4 as high-risk for hospital-associated VTE, meriting VTE prophylaxis) [15].

To characterize inpatient VTE prophylactic anticoagulation, abstractors reviewed prescribing data and clinical notes. Reviewers defined anticoagulant medications as prophylaxis (rather than treatment) if they were prescribed for at least 50% of hospital days prior to any diagnosis of VTE. The drug, dose, route and frequency were collected. We categorized prophylactic anticoagulation into 3 strategies based on the agent, timing and dose (cross-referencing with patient weight): no pharmacologic prophylaxis, standard prophylaxis (e.g. enoxaparin 40mg subcutaneous daily or non-weight-based standard high-risk prophylaxis based on bariatric and orthopedic surgery protocols, e.g. enoxaparin 30 or 40mg subcutaneously every 12 hours), intensified prophylaxis (e.g. 0.5mg/kg enoxaparin subcutaneously every 12 hours or 1mg/kg enoxaparin subcutaneously daily), or therapeutic dose given as prophylaxis (e.g. 1mg/kg enoxaparin subcutaneously every 12 hours or unfractionated intravenous heparin drip). Post-discharge pharmacologic prophylaxis was defined as anticoagulants prescribed at discharge for patients without VTE during hospitalization.

We collected laboratory data on D-dimer at presentation, peak D-dimer and institutional D-dimer upper limit of normal (ULN). Based on biologic and clinical relevance, we prospectively categorized D-dimer according to whether it was below the ULN (<ULN) for a given site, between 1-6-fold the ULN (1-6x ULN), or greater than or equal to 6-fold the ULN (>6x ULN) [16].

Diagnosis of venous and arterial thrombotic and bleeding outcomes was made during routine clinical care. To identify thrombotic and bleeding events, abstractors reviewed all relevant documentation from admission up to 30 days post-discharge, including external hospital records for post-discharge events. VTE was defined as DVT or PE diagnosed radiographically (for PE, CT chest imaging, formal echocardiogram or point-of-care echocardiogram, or V/Q scan; for DVT, formal ultrasound or point-of-care ultrasound) or clinically (without confirmatory imaging). For analyses with VTE as the outcome and prophylaxis as the predictor, we excluded VTEs diagnosed within 48 hours of admission to analyze events likely to be affected by inpatient prophylaxis exposure (versus VTEs likely already present on admission). ATE (myocardial infarction, ischemic stroke, and other systemic thromboembolism) was defined as radiologic, operative, or procedural evidence of acute ATE, or clinician documentation of an acute ATE. Bleeding was defined according to International Society for Thrombosis and Hemostasis definitions of major bleeding (MB) and clinically relevant non-major bleeding (CRNMB) [17, 18]. The ISTH defines MB as hemorrhage that is fatal, in a critical area or organ or bleeding that causes a drop in hemoglobin level of 2g/dL or more or leading to transfusion of 2 or more units of packed red blood cells. CRNMB is defined by the ISTH as any sign or symptom of hemorrhage that does not fit the criteria for the ISTH definition of major bleeding but does meet at least one of the following criteria: 1) requiring medical intervention by a healthcare professional, 2) leading to hospitalization or increased level of care, 3) prompting a face-to-face evaluation.

### Statistical analysis

We estimated a sample size of 1000 patients to provide 80% power to detect an 8% reduction in VTE rates between 2 dominant prophylactic strategies. We report results as mean ± standard deviation, median (interquartile range [IQR]), or proportion (percentage) as appropriate. The primary outcome was VTE (PE and DVT); secondary outcomes included PE, DVT, ATE, bleeding (MB and CRNMB), MB and CRNMB.

To examine associations between predictors and outcomes, we fit separate unadjusted and multivariable (adjusted) logistic regression models that accounted for clustering by site. We adjusted for age, race/ethnicity, sex, body mass index (BMI) and clinical risk factors that impart a high risk for severe COVID-19: chronic lung disease, cardiovascular disease, immunocompromise, diabetes mellitus, end-stage renal disease on dialysis and cancer [19]. Laboratory parameters were not adjusted for one another. We also performed pre-specified subgroup analyses to examine the effect of intensity of VTE prophylaxis on development of VTE by stratifying within patients of similar observable VTE risk, including admission to the ICU, intubation, and by baseline and peak D-dimer level.

To address confounding by indication, we used inverse probability weighting (IPTW), a robust method that weighs patients using the inverse of their probability of receiving a given treatment (in this case, VTE prophylaxis intensity). We chose to use IPTW over other methods that account for confounding by indication (like propensity score matching) because it allows for analysis of all eligible patients and for comparison of more than two treatments [20–22]. To perform IPTW, we: (1) estimated a patient’s probability of receiving a given VTE prophylaxis intensity by fitting a separate logistic regression model with VTE prophylaxis intensity as the outcome regressed upon patient characteristics that predict both prophylaxis intensity and the primary outcome (VTE) (age, race/ethnicity, sex, body mass index (BMI), chronic lung disease, cardiovascular disease, immunocompromise, diabetes mellitus, end-stage renal disease on dialysis, cancer, admission to the ICU and intubation), (2) assigned each patient a weight based on the inverse of the predicted probabilities generated from this separate logistic regression model, (3) incorporated these weights into multivariable logistic regression models.

We calculated a cumulative kappa (κ) for key primary and secondary outcome variables (PE, DVT, MB, CRNMB) by measuring the inter-rater reliability within site and then determining the average κ across 33 sites. We performed all analyses using STATA (Version 16.1, College Station, TX). STROBE statement checklist can be found in **S2 Table in S1 File.**

## Results

### Cohort characteristics

The cohort was comprised of 1121 patients admitted with COVID-19 between February 2, 2020 and December 31, 2020 to 33 US academic medical centers (**Table 1)**. Twelve sites (36%) reviewed 20 patients, 15 sites (45%) reviewed 21–40 patients, and 6 (18%) reviewed more than 40 patients (range 20–59). The average κ across sites was 0.73, suggesting moderate cumulative inter-rater reliability [23].

**Table 1:** Patient characteristics of 1121 hospitalized patients with COVID-19 at 33 US academic medical centers.

**Characteristics**	**All patients**	**No prophylaxis**	**Standard prophylaxis**	**Intensified prophylaxis**	**Treatment dose prophylaxis**
** **	**n = 1121**	**n = 156**	**n = 754**	**n = 17**	**n = 189**
Age, mean; SD	60; 18	56; 21	60; 17	65; 15	65; 17
Female sex, n (%)	522 (47)	80 (51)	357 (47)	5 (29)	80 (42)
Race ethnicity					
Asian, n (%)	55 (5.0)	8 (5)	42 (5.6)	1 (5.9)	4 (2.1)
Black/African-American, n (%)	355 (32)	44 (28)	246 (33)	4 (24)	61 (32)
Hispanic, n (%)	268 (24)	36 (23)	201 (27)	2 (12)	29 (15)
White, n (%)	375 (33)	61 (39)	224 (30)	10 (59)	79 (42)
Other, n (%)	65 (5.8)	7 (4.5)	41 (5.4)	0 (0)	16 (8.5)
**Anticoagulant agent prior to hospitalization**					
Any anticoagulant, n (%)	122 (10)	12 (7.7)	14 (1.9)	0 (0)	96 (51)
Warfarin, n (%)	33 (2.9)	5 (3.2)	3 (0.40)	0 (0)	25 (13)
DOAC, n (%)	79 (6.9)	6 (3.9)	8 (1.1)	0 (0)	65 (34)
LMWH, n (%)	10 (0.87)	1 (0.64)	3 (0.40)	0 (0)	6 (3.1)
**Antiplatelet agent prior to hospitalization**					
Any antiplatelet, n (%)	303 (27)	36 (23)	213 (28)	6 (35)	48 (25)
Aspirin, n (%)	255 (22)	26 (17)	183 (24)	5 (28)	41 (21)
Clopidogrel, n (%)	40 (3.5)	9 (5.8)	24 (3.2)	1 (5.9)	6 (3.2)
Prasugrel, n (%)	2 (0.17)	0 (0)	1 (0.13)	0 (0)	1 (0.13)
Ticagrelor, n (%)	4 (0.35)	1 (0.64)	3 (0.40)	0 (0)	0 (0)
Dipyrimadole, n (%)	1 (0.09)	0 (0)	1 (0.13)	0 (0)	0 (0)
Cilostazol, n (%)	1 (0.09)	0 (0)	1 (0.13)	0 (0)	0 (0)
**Risk factors**					
Active cancer, n (%)	61 (5.4)	14 (9)	35 (4.6)	0 (0)	12 (6.3)
Diabetes, n (%)	399 (35)	43 (28)	272 (36)	4 (24)	80 (42)
Prior VTE, n (%)	68 (6.1)	10 (6.4)	20 (2.7)	0 (0)	38 (20)
Prior thrombophilia, n (%)	10 (0.89)	1 (0.64)	5 (0.66)	0 (0)	4 (2.1)
Recent (<30d) trauma or surgery, n (%)	45 (4.0)	14 (9)	26 (3.4)	0 (0)	5 (2.6)
ESRD on HD, n (%)	56 (5.0)	5 (3.2)	40 (5.2)	0 (0)	11 (5.8)
Pre-existing lung disease, n (%)	211 (19)	28 (18)	141 (19)	4 (22)	38 (20)
Pre-existing immunosuppression, n (%)	112 (10)	17 (11)	71 (9.3)	2 (11)	22 (12)
**Risk factors (continued)**	**All patients**	**No prophylaxis**	**Standard prophylaxis**	**Intensified prophylaxis**	**Treatment dose prophylaxis**
** **	**n = 1121**	**n = 156**	**n = 754**	**n = 17**	**n = 189**
Pre-existing heart failure, n (%)	157 (14)	10 (6.4)	91 (12)	1 (5.6)	55 (29)
Pre-existing arterial vascular disease, n (%)	203 (18)	21 (13)	119 (16)	4 (22)	59 (31)
Pre-existing atrial fibrillation, n (%)	118 (11)	8 (5.1)	46 (6.1)	1 (5.6)	63 (33)
Ongoing prothrombotic hormone use, n (%)	13 (1.2)	4 (2.6)	7 (0.93)	0 (0)	2 (1.1)
BMI >/ = 30, n (%)	555 (49)	68 (44)	380 (50)	13 (72)	94 (49)
**Illness severity**					
Days from symptoms to admission, median (IQR)	6 (2,9)	6 (1,11)	5 (2,9)	7 (6,12)	7 (2,10)
Ward, n (%)	738 (66)	122 (78)	502 (66)	7 (39)	107 (56)
ICU, n (%)	383 (34)	34 (22)	254 (34)	11 (61)	84 (44)
Intubated, n (%)	232 (21)	12 (7.7)	155 (21)	5 (28)	60 (31)
Oxygen without intubation, n (%)	573 (50)	54 (35)	412 (55)	10 (59)	97 (51)
Hospital LOS, median (IQR)	8 (3,17)	4 (2,8)	7 (4,14)	16 (6,25)	9 (5,20)
ICU LOS, median (IQR)	7 (3,14)	2 (1,5)	8 (3,17)	9 (5,27)	11 (4,23)
Peak D-dimer, median (IQR)	1535 (745,4101)	1880 (780,3230)	1420 (700,3610)	3690 (1500,7129)	2160 (910,7325)
Peak Creatinine, median (IQR)	1.1 (0.8, 1.8)	1 (0.8, 1.6)	1.1 (0.8,1.6)	1.1 (0.9, 1.3)	1.4 (1.0,3)
Padua score, median (IQR)	6 (5,6)	5 (5,6)	6 (5,6)	6 (5,6)	6 (6,7)
Death during hospitalization, n (%)	137 (12)	19 (12)	76 (10)	2 (11)	40 (21)

Abbreviations- SD = standard deviation, VTE = venous thromboembolism, d = days, BMI = body mass index, IQR = interquartile range, ICU = intensive care unit, LOS = length of stay

Overall, mean age was 60 ± 18, 47% were female, 33% identified as White and 32% as Black. Median Padua score was 6 (IQR 5,6). A median of 6 days (IQR 2,9) had elapsed between symptom onset and hospitalization. One-third were admitted to the ICU, and 21% required intubation. Median length of stay was 7 days (IQR 3,14). One-hundred thirty-seven patients (12%) died during hospitalization. The most common comorbid conditions were diabetes (35%), pre-existing lung disease (19%) and pre-existing vascular disease (18%); six percent had a prior VTE, 5% had cancer, and half were obese. Prior to admission, 122 patients (11%) were prescribed anticoagulants and 303 patients (27%) antiplatelet agents for chronic conditions (e.g. atrial fibrillation). Patient characteristics for the subgroup of patients admitted to the intensive care unit are provided in S5 Table in S1 File.

### Practice patterns for VTE prophylaxis

Eight-six percent (965) of hospitalized patients received pharmacologic VTE prophylaxis (**Table 2**). Those not receiving prophylaxis had younger mean age (56 ± 21), fewer were in the ICU (20%) or intubated (8%), and they had lower median Padua scores (median 5, IQR 5,6). Two-thirds received enoxaparin subcutaneously, 18% received unfractionated heparin subcutaneously and 6% intravenously, and 7% received direct oral anticoagulants. Fifty percent (559) received standard dosing, 17% (195) standard high-risk dosing, 2% (17) intensified dosing, and 17% (189) therapeutic dosing. Thirty-five patients (3%) without VTE received post-discharge pharmacologic prophylaxis.

**Table 2:** Practice patterns for VTE prophylaxis and diagnosis.

Form of VTE prophylaxis	Data (n = 1121)
Any pharmacologic, n (%)	965 (86)
DOAC, n (%)	68 (7.0)
Enoxaparin, n (%)	636 (66)
UFH (IV), n (%)	62 (6.4)
UFH (SQ), n (%)	175 (18)
Other, n (%)	24 (2.5)
**Dose of VTE prophylaxis**	** **
Standard dosing, n (%)	559 (50)
Standard high-risk dosing, n (%)	195 (17)
Intensified (weight-based) dosing, n (%)	17 (1.5)
Therapeutic dosing, n (%)	189 (17)
**Post-discharge prophylaxis**	** **
Pharmacologic, n (%)	38 (3.4)
**Diagnostic test**	
**Inpatient pulmonary embolism**	
Computed topography pulmonary embolism	41 (3.6)
R heart dysfunction on formal echocardiogram	3 (0.26)
R heart dysfunction on point-of-care ultrasound	3 (0.26)
**Inpatient deep vein thrombosis**	
Ultrasound	45 (4)
CT scan	6 (0.54)

Abbreviations- VTE = venous thromboembolism, DOAC = direct oral anticoagulant, UFH = unfractionated heparin, IV = intravenous, SQ = subcutaneous, R = right, CT = computed topography

### Practice patterns for VTE diagnosis

No patients in our sample had VTE diagnosed solely on clinical grounds (**Table 2**). Forty-three patients (3.8%) were diagnosed with PE using CTPE, 3 (0.26%) with formal transthoracic echocardiogram and 3 (0.26%) with point-of-care cardiac ultrasound. DVT was diagnosed by formal DVT ultrasound in 45 patients (4%) and by CT scan of the leg veins in 6 patients (0.52%).

### Venous and arterial thromboembolism

Eighty-nine patients had a VTE at any time during hospitalization: 47 had PE (4.2%), 51 had DVT (4.6%), and 9 (0.8%) had both PE and DVT (**Table 3**). To analyze events most likely to be affected by prophylaxis strategy, we excluded VTE events diagnosed within 48 hours of admission—24 PEs and 12 DVTs **(S3 Table in S1 File**). Of the remaining 23 (2.1%) patients with PE diagnosed at least 48 hours post-admission, median time to diagnosis was 10 days (IQR 5,16). Of the 39 patients with DVT, median time to diagnosis was 11 days (IQR 6,21). Five patients (0.44%) had both DVT and PE diagnosed at least 48 hours post-admission.

**Table 3:** Venous and arterial thromboembolism and bleeding events.

Inpatient thrombosis	Total events, n/1121 (%)	>48H after admission	<48H after admission
Pulmonary embolism, n (%)	47 (4.2)	23 (2.1)	24 (2.1)
Peri-PE hypotension, n (%)	9 (0.80)	8 (0.71)	1 (0.09)
Right heart strain, n (%)	15 (1.3)	9 (0.80)	6 (0.54)
Segmental only, n (%)	14 (1.2)	5 (0.45)	9 (0.80)
Days from admission to PE, median (IQR)	1 (0,9)	10 (5,16)	0 (0,1)
Deep vein thrombosis, n (%)	51 (4.6)	39 (3.5)	12 (1.1)
Upper extremity, n (%)	20 (1.8)	18	2
Lower extremity, n (%)	30 (2.7)	21	9
Other, n (%)	2 (0.18)	1	1
CVC-associated, n (%)	11 (0.96)	9	2
Days from admission to DVT, median (IQR)	8 (2,18)	11 (6,21)	1 (0,1)
Baseline D-dimer in those with VTE, median (IQR)	1875 (700,6096)	986 (550,1830)	990 (560,1840)
Peak D-dimer in those with VTE, median (IQR)	7322 (2803,17,155)	1498 (733,4000)	1438 (701,3610)
Arterial thrombosis, n (%)	23 (2.1)	9 (0.80)	14 (1.2)
CVA	13 (1.2)	5	8
MI	7 (0.61)	2	5
Systemic arterial embolism	3 (0.26)	2	1
Days from admission to ATE, median (IQR)	1 (0,10)	10 (8,18)	0 (0,1)
**Post-discharge thrombosis**			
Post-discharge PE, n (%)	4 (0.35)		
Post-discharge DVT, n (%)	2 (0.18)		
Days from discharge to VTE, median (IQR)	8 (6, 30)		
Post-discharge ATE, n (%)	0 (0)		
**Inpatient bleeding**			
Major bleed, n (%)	46 (4.1)		
MB associated with therapeutic AC, n (%)	23 (50)		
CRNMB, n (%)	46 (4.1)		
CRNMB with therapeutic AC, n (%)	25 (54)		
Number of pRBC units transfused, median (IQR)	2 (0,7)		
**Post-discharge bleeding**			
Post-discharge MB, n (%)	6 (0.52)		
Post discharge MB with prophylactic AC, n (%)	0 (0)		
Post-discharge MB with therapeutic AC, n (%) Days from discharge to MB, median (IQR)	2 (30) 20 (18,27)		

Abbreviations- H = hours, PE = pulmonary embolism, IQR = interquartile range, CVC = central venous catheter, DVT = deep vein thrombosis, CVA = cerebrovascular accident, MI = myocardial infarction, ATE = arterial thromboembolism, MB = major bleed, AC = anticoagulation, CRNMB = clinically-relevant non-major bleed, pRBC = packed red blood cells

Four patients (0.35%) had PE and 2 patients (0.18%) had DVT post-discharge, one of whom had a VTE during hospitalization and was prescribed therapeutic anticoagulation at discharge; VTE was diagnosed at a median of 8 days (IQR 6,30) post-discharge.

Twenty-three patients had arterial thrombosis at a median of 1 day (IQR 0,10) after admission—13 (1.1%) with CVA, 7 (0.61%) with MI, and 3 (0.26%) with systemic arterial embolism. No patients experienced an arterial thrombosis post-discharge. VTE events in the subgroup of patients admitted to the ICU are presented in **S6 Table in S1 File.**

### Bleeding

There were 84 patients (7.5%) with bleeding events during hospitalization; 33% occurred on therapeutic dosing of anticoagulants as prophylaxis or treatment (**Table 3**). Forty-six (4.1%) MB events occurred during hospitalization; twenty-three of these (50%) occurred on therapeutic dosing. Forty-six (4.1%) CRNMB events occurred, 25 of which (54%) occurred on therapeutic dosing. Eight patients (0.71%) had both MB and CRNMB. Among patients who received therapeutic dosing as prophylaxis, 15% had bleeding of any kind, 5% had a MB, and 12% had a CRNMB. Six patients (0.52%) had MB at a median of 20 days (IQR 18–27) post-discharge, 2 of whom (30%) were prescribed therapeutic anticoagulation for diagnosed VTE and none of whom was prescribed post-discharge prophylaxis. Bleeding events in the subgroup of patients admitted to the ICU are presented in **S6 Table in S1 File.**

### Prophylaxis strategy and association with thrombosis

**Fig 1:**
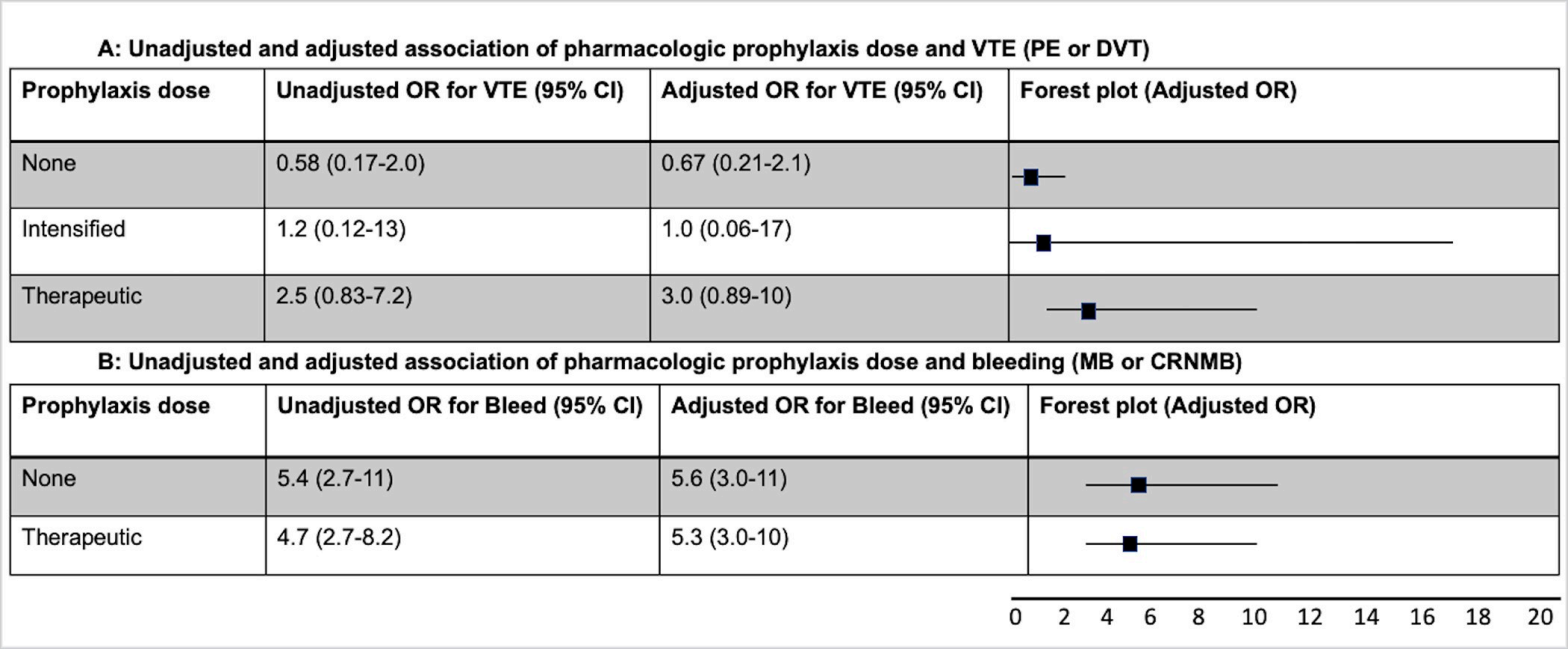
1A and 1B: Unadjusted and adjusted association of pharmacologic prophylaxis dose and venous thromboembolism or bleeding. Unadjusted and adjusted odds ratios (ORs) and Forest plot (with adjusted OR) are shown according to dose of pharmacologic VTE prophylaxis. All ORs are in comparison to standard VTE pharmacologic prophylaxis dosing. In Fig 1A, ORs and Forest plot for venous thromboembolism (pulmonary embolism or deep vein thrombosis) are shown. In Fig 1B, ORs and Forest plot for bleeding (major bleeding or clinically relevant non-major bleeding) are shown. Abbreviations- OR = odds ratio, VTE = venous thromboembolism, PE = pulmonary embolism, DVT = deep vein thrombosis, CI = confidence interval, MB = major bleeding, CRNMB = clinically relevant non-major bleeding.

In multivariable analysis, compared to standard prophylaxis, the odds of VTE diagnosis 48 hours after admission were 0.67 (95% CI 0.21–2.1) with no pharmacologic prophylaxis, 1.0 (95% CI 0.06–17) with intensified dosing, and 3.0 (95% CI 0.89–10) with therapeutic dosing (**Fig 1A**). No PEs occurred on intensified prophylaxis dosing, but odds of pulmonary embolism were 1.0 (95% CI 0.20–5.1) with no prophylaxis and 4.4 (95% CI 2.0–9.5) with therapeutic dosing. The odds of DVT were 0.80 (95% CI 0.18–3.4) on no prophylaxis, 1.3 (95% CI 0.06–27) on intensified dose, and 2.6 (95% CI 0.45–15) on therapeutic dose. Results were similar for VTE occurring within 48 hours of admission and in subgroup analyses according to ICU status, intubation, and D-dimer level **(S3 & S4 Tables in S1 File).**

Using inverse probability weighting, the odds of VTE were 1.2 (95% CI 0.28–5.1) with no pharmacologic prophylaxis, 2.5 (95% CI 0.24–27) with intensified dosing, and 2.9 (95% CI 0.81–10) with therapeutic dosing. No PEs occurred on intensified prophylaxis dosing, but the odds of pulmonary embolism were 2.9 (95% CI 0.63–14) with no prophylaxis and 3.9 (95% CI 1.7–8.9) with therapeutic dosing. Odds of DVT were 1.5 (95% CI 0.23–9.3) on no prophylaxis, 3.1 (95% CI 0.23–42) on intensified dose, and 2.5 (95% CI 0.43–14) on therapeutic dose.

### Prophylaxis strategy and bleeding

No patients experienced a bleeding event on intensified prophylaxis, so multivariable analysis is available for no prophylaxis and therapeutic dosing compared to standard. Odds of bleeding on no prophylaxis were 5.6 (95% CI 3.0–11) versus 5.3 (95% CI 3.0–10) for therapeutic (**Fig 1B**). Odds of MB were 3.8 (95% CI 1.8–8.3) for no prophylaxis and 2.1 (95% CI 0.95–4.5) for therapeutic. Odds of CRNMB were 4.8 (95% CI 1.7–14) for no prophylaxis and 7.6 (95% CI 3.0–19) for therapeutic.

With inverse probability weighting, odds of bleeding on no prophylaxis were 6.8 (95% CI 3.1–14) versus 4.7 (95% CI 2.6–8.6) with therapeutic dosing. For MB, the odds were 4.6 (95% CI 1.8–11) for no prophylaxis versus 1.6 (95% CI 0.63–4) for therapeutic. The odds of CRNMB were 4.9 (95% CI 1.6–15) for no prophylaxis versus 7.0 (95% CI 2.9–17) for therapeutic.

### D-dimer, thrombosis and prophylaxis

D-dimer was measured for 816 patients (73%). Median baseline D-dimer during hospitalization was 1000 mcg/L (fibrin equivalent units, FEU) (IQR 560, 1880), drawn at a mean of 1 ± 4 days post-admission; median peak D-dimer was 1535 mcg/L (IQR 745, 4101), drawn at a mean of 4 ± 7 days post-admission. Median baseline D-dimer among patients with VTE was 1875 mcg/L (IQR 700, 6096); median peak D-dimer was 7322 mcg/L (IQR 2803, 17,155).

Elevation in baseline D-dimer >6x ULN was associated with increased odds of VTE (OR 2.4, 95% CI 1.3–9.4), while elevation 1-6x ULN was not (OR 1.3, 95% CI 0.55–4.4). Elevation in peak D-dimer >6x ULN was associated with increased odds of VTE (OR 4.5, 95% CI 1.5–13.7), while elevation 1-6x ULN was not (OR 0.94, 95% CI 0.27–3.3).

Baseline elevation in D-dimer >6x ULN was associated with increased odds of receipt of intensified-dose or therapeutic anticoagulation (OR 3.5, 95% CI 1.8–6.8); peak elevation in D-dimer >6x ULN was associated with increased odds of receiving intensified-dose or therapeutic anticoagulation (OR = 3.6, 95% CI 2.1–6.1).

## Discussion

This multicenter study adds key information on VTE practice patterns and outcomes in the early phase of the pandemic. Rates of thrombosis in this cohort were lower than initial reports from Asia and Europe but similar to other concurrent studies published in the US: PE occurred in 4.2%, DVT occurred in 4.6%, ATE occurred in 2.1%, and post-discharge VTE occurred in 0.54% [2–5, 19]. Only marked elevation in baseline or peak D-dimer (>6xULN) was associated with increased odds of VTE. Bleeding rates were higher than published reports: 7.5% had any bleeding, 4.1% had MB, 4.1% had CRNMB, and 0.52% had bleeding after discharge [2, 19, 24]. Bleeding rates for therapeutic dose anticoagulation given as prophylaxis were 15% for bleeding, 5% for MB and 12% for CRNMB. Overall, we believe that our data accurately reflect the bleeding risk of high-dose anticoagulation in patients with COVID-19 in a real-world population, which likely exceeds that seen in clinical trials that excluded those at high risk of bleeding. However, our findings of elevated thrombosis risk associated with full-dose anticoagulation likely reflect empiric treatment of suspected VTE prior to confirmed VTE diagnosis, rather than high-intensity anticoagulation leading to VTE.

We note that the practice we observed of pre-emptive prophylaxis escalation is not unexpected. During our study period from February through December 2020, there were mounting reports of VTE risk but little evidence to guide prevention and treatment strategies, and some experts and hospital guidelines advocated full-dose prophylactic anticoagulation in COVID-19 patients with clinical instability [6, 25]. We found that 86% of patients received VTE prophylaxis in our study, a proportion similar to that seen in other observational studies conducted over similar time frames in the US and internationally [4, 26]. In addition, we found that nearly 1 in 5 patients received either intensified or therapeutic dose anticoagulation given as prophylaxis, a practice that came at the cost of increased bleeding. Compared to standard dosing, patients receiving therapeutic dosing had greater than 5 times the odds of bleeding. One-third of bleeding among hospitalized patients and one-third of bleeding post-discharge occurred on therapeutic anticoagulation. Since then, the optimal dose of prophylactic anticoagulation for patients hospitalized with COVID-19 has been evaluated in several randomized trials published in 2021. In general, these data support that therapeutic anticoagulation is associated with improved outcomes in hospitalized patients with COVID-19 who are not critically ill, whereas those in the ICU do not benefit and have increased bleeding risk. In addition, there appears to be no benefit to any dosing between prophylactic or therapeutic as was employed during our study period. The higher bleeding rates we observed in our study exceeding those reported in these clinical trials highlights the value of reporting on outcomes in actual clinical practice.

We found a 3-fold increase in the odds of VTE with therapeutic dose and reduced odds of VTE with no prophylaxis compared to standard dose VTE prophylaxis. Although we attempted to account for treatment allocation bias using IPTW, these findings likely reflect residual confounding by indication [20–22]. Because chart reviewers only recorded medications given specifically as VTE prophylaxis before a diagnosis of VTE, it is unlikely that therapeutic anticoagulation given as treatment for known, chronic thrombotic conditions or newly diagnosed VTE was miscategorized as prophylaxis. In further tests of robustness, the association was present when restricting to thromboses that occurred after at least 48 hours of VTE prophylaxis exposure and across subgroups. Patients who were not admitted to the ICU, not intubated and who had D-dimer >6xULN had the highest odds of VTE while on therapeutic dose anticoagulation given as prophylaxis. Thus, as noted above, we conclude that clinicians pre-emptively escalated VTE prophylaxis dosing to therapeutic intensity based on suspicion of thrombosis. Thrombosis was then subsequently confirmed with diagnostic testing in a large percentage of patients. The finding that marked elevation in D-dimer was associated with either intensified or therapeutic dose anticoagulation given as prophylaxis supports this explanation. Given these limitations, we cannot draw conclusions about the efficacy of higher-than-standard prophylaxis dosing for prevention of VTE.

### Strengths and limitations

A major strength of this study was our use of detailed chart review by clinicians. This allowed for granular and accurate information on prophylaxis strategies, clinical decision-making, and thrombotic and bleeding events, all of which can be inadequately captured by traditional administrative coding. There were no missing data for primary or secondary outcomes or covariates. Our multi-hospital sample included patients in disparate geographic locations and practice settings.

The limitations of this study relate to its retrospective observational nature. As a study of routine clinical care, patients were not randomly assigned to groups, meaning it is possible that outcomes may have been influenced by factors leading to preferential use of one anticoagulant strategy over another, such as variations in local practice, intercurrent health events, or hospital shortages. As above, we attempted to account for this with robust methods, but the findings of higher VTE rates in those receiving full-dose anticoagulation strongly suggest residual confounding by indication.

Data on post-discharge outcomes may have been missed. Although abstractors screened for outcomes in outside hospital records, some bleeding and thrombotic events may not have been contained in these records, such as those that were too minor to lead to medical attention, fatal, or outside the abstractor’s healthcare systems. Longer follow-up or screening imaging may have revealed additional events. Some patients were missing laboratory or other information on predictors. Because our analysis was restricted to patients admitted to academic medical centers, our results may not be as broadly generalizable.

## Conclusions

Our findings confirm that COVID-19 is associated with an increased risk of thrombosis and bleeding. In the first 10 months of the pandemic, as clinicians rushed to meet the demands of a rapidly advancing pandemic, use of higher-than-standard pharmacologic VTE prophylaxis was common and was associated with increased risk of VTE, as well as bleeding. Overall, our observational findings suggest bleeding rates in clinical practice that exceed those seen in clinical trials. Our findings also suggest that clinicians astutely started full-dose anticoagulation as “prophylaxis” in patients who ultimately were found to have thrombosis.

## Supporting information

S1 File Supplement to “Venous thromboembolism (VTE) prevention and diagnosis in COVID-19: Practice patterns and outcomes at 33 hospitals”. This supplement includes: S1 Table, S1 Fig, S2—S6 Tables.
(DOCX)
