# Supplementary material for: Venous thromboembolism (VTE) prevention and diagnosis in COVID-19: Practice patterns and outcomes at 33 hospitals
Source: PLoS One. 2022 May 5;17(5):e0266944. doi: 10.1371/journal.pone.0266944 (PMC9071149; doi:10.1371/journal.pone.0266944)
Supplement: S1 File — This supplement includes: S1 Table, S1 Fig, S2—S6 Tables. (DOCX) [file pone.0266944.s001.docx]

**Venous Thromboembolism (VTE) Prevention and Diagnosis in COVID-19: Practice Patterns and Outcomes at 33 Hospitals**

Anna L. Parks, MD (1), Andrew D. Auerbach, MD (2), Jeffrey L. Schnipper, MD, MPH (3), Amanda Bertram, MS (4), Sun Y. Jeon, MS PhD (5) Bridget Boyle (4), Margaret C. Fang, MD, MPH (2), Shrirang M. Gadrey, MBBS, MPH (6), Zishan K. Siddiqui, MD (4), Daniel J. Brotman, MD (4) and the Hospital Medicine Reengineering Network (HOMERuN)

1. Division of Hematology and Hematologic Malignancies, Department of Medicine, University of Utah, Salt Lake City, UT, USA
2. Division of Hospital Medicine, Department of Medicine, University of California, San Francisco, San Francisco, CA, USA
3. Division of General Internal Medicine, Brigham and Women's Hospital, Boston, MA, USA
4. Division of Hospital Medicine, Department of Medicine, Johns Hopkins School of Medicine, Baltimore, MD, USA.
5. Division of Geriatrics, University of California, San Francisco and San Francisco VA Medical Center, San Francisco, CA
6. Division of General, Geriatric, Palliative and Hospital Medicine, University of Virginia, Charlottesville, VA, USA

Table of Contents

[S1 Table 1: List of HOMERuN collaborative participating institutions 2](#_Toc98503841)

[S1 Figure 1: Cohort flow diagram 5](#_Toc98503842)

[S2 Table 2: STROBE statement checklist 6](#_Toc98503843)

[S3 Table 3: Adjusted Odds Ratios for total VTE, VTE >48H after admission and VTE <48H after admission 8](#_Toc98503844)

[S4 Table 4: Prophylaxis dose subgroup analyses 9](#_Toc98503845)

[S5 Table 5: Patient characteristics of subgroup of 383 patients admitted to the intensive care unit with COVID-19 at 33 US academic medical centers 10](#_Toc98503846)

[S6 Table 6: Venous and arterial thromboembolism and bleeding events in subgroup of 383 patients admitted to the intensive care unit with COVID-19 at 33 US academic medical centers 12](#_Toc98503847)

## S1 Table 1: List of HOMERuN collaborative participating institutions

| **Hospital name** | **Region Division** | **Beds** | **Type** |
| --- | --- | --- | --- |
| Barnes Jewish Hospital/Washington University St. Louis | West North Central | 1,400 | Academic |
| Baylor Scott & White Medical Center Temple | West South Central | 636 | Academic |
| Baystate Medical Center | New England | 716 | Academic |
| Beth Israel Deaconess Medical Center | New England | 673 | Academic |
| Brigham and Women's Hospital | New England | 793 | Academic |
| Cedars-Sinai Medical Center | Pacific | 886 | Academic |
| ChristianaCare | South Atlantic | 1,227 | Academic |
| Cleveland Clinic | East North Central | 1,300 | Academic |
| Dartmouth-Hitchcock Medical Center | New England | 422 | Academic |
| Dell Seton Medical Center/UT Austin | West South Central | 430 | Academic |
| Denver Health and Hospitals Authority | Mountain | 525 | Safety Net |
| Emory University Hospital | South Atlantic | 733 | Academic |
| Evanston Hospital/NorthShore | East North Central | 354 | Academic |
| Froedtert Hospital/Medical College of Wisconsin | East North Central | 607 | Academic |
| Harborview Medical Center | Pacific | 413 | Safety Net |
| Johns Hopkins Hospital | South Atlantic | 1,162 | Academic |
| Johns Hopkins Bayview Medical Center | South Atlantic | 420 | Safety Net |
| Maine Medical Center | New England | 637 | Academic |
| Massachusetts General Hospital | New England | 999 | Academic |
| Mayo Clinic Rochester | West North Central | 2,059 | Academic |
| Miriam Hospital | New England | 247 | Academic |
| Mount Sinai Hospital | Middle Atlantic | 1,141 | Academic |
| Northwestern Memorial Hospital | East North Central | 894 | Academic |
| Ohio State University Wexner Medical Center | East North Central | 1,397 | Academic |
| Oregon Health & Science University | Pacific | 556 | Academic |
| Penn Medicine (Hospital of the University of Pennsylvania and Penn Presbyterian Medical Center) | Middle Atlantic | 776 | Academic |
| Robert Wood Johnson University Hospital/Rutgers | Middle Atlantic | 965 | Academic |
| Stanford | Pacific | 605 | Academic |
| Tulane Hospital and University Medical Center | West South Central | 235 | Academic |
| UC San Diego | Pacific | 808 | Academic |
| UC San Francisco | Pacific | 796 | Academic |
| UCLA Ronald Reagan Hospital | Pacific | 520 | Academic |
| UK HealthCare | East South Central | 945 | Academic |
| University of Chicago | East North Central | 811 | Academic |
| University of Colorado Denver Anschutz Medical Campus | Mountain | 400 | Academic |
| University of Florida Shands Hospital | South Atlantic | 1,162 | Academic |
| University of Iowa Hospitals and Clinics | West North Central | 811 | Academic |
| University of Miami Hospital | South Atlantic | 560 | Academic |
| University of Michigan | East North Central | 1,000 | Academic |
| University of Missouri-Columbia Hospital | West North Central | 247 | Academic |
| University of Nebraska Medical Center / Nebraska Medicine | West North Central | 800 | Academic |
| University of North Carolina at Chapel Hill | South Atlantic | 950 | Academic |
| University of Pittsburgh Medical Center Presbyterian | Middle Atlantic | 680 | Academic |
| University of Vermont Medical Center | New England | 562 | Academic |
| University of Washington Medical Center | Pacific | 570 | Academic |
| University of Wisconsin Hospital and Clinics | East North Central | 505 | Academic |
| UW-Northwest | Pacific | 281 | Academic |
| Vanderbilt University Medical Center | East South Central | 1,019 | Academic |
| Wake Forest Baptist | South Atlantic | 1,535 | Academic |
| Weill Cornell | Middle Atlantic | 862 | Academic |
| Yale New Haven Hospital | New England | 1,541 | Academic |
| Zuckerberg San Francisco General Hospital | Pacific | 397 | Safety Net |
| Baystate Health | New England | 724 | Academic |
| Boston University | New England | 496 | Academic |
| BSWH University Medical Center-Dallas | West South Central | 1,025 | Academic |
| Columbia University | Middle Atlantic | 738 | Academic |
| Duke | South Atlantic | 979 | Academic |
| Louisiana State University | West South Central | 446 | Academic |
| Loyola University | East North Central | 254 | Academic |
| Medical University of South Carolina | South Atlantic | 1,600 | Academic |
| Montefiore | Middle Atlantic | 1,491 | Academic |
| NYU Langone | Middle Atlantic | 725 | Academic |
| Penn State University | Middle Atlantic | 548 | Academic |
| Pennington Biomedical Research Center | West South Central |  |  |
| Rush University | East North Central | 697 | Academic |
| UC Davis | Pacific | 617 | Academic |
| UC Irvine | Pacific | 422 | Academic |
| University of Illinois | East North Central | 462 | Academic |
| University of Kansas Medical Center | West North Central | 910 | Academic |
| University of Minnesota | West North Central | 743 | Academic |
| University of Southern California | Pacific | 600 | Academic |
| University of Texas Houston | West South Central | 514 | Academic |
| University of Utah | Mountain | 527 | Academic |
| UT Southwestern | West South Central | 640 | Academic |

## S1 Figure 1: Cohort flow diagram


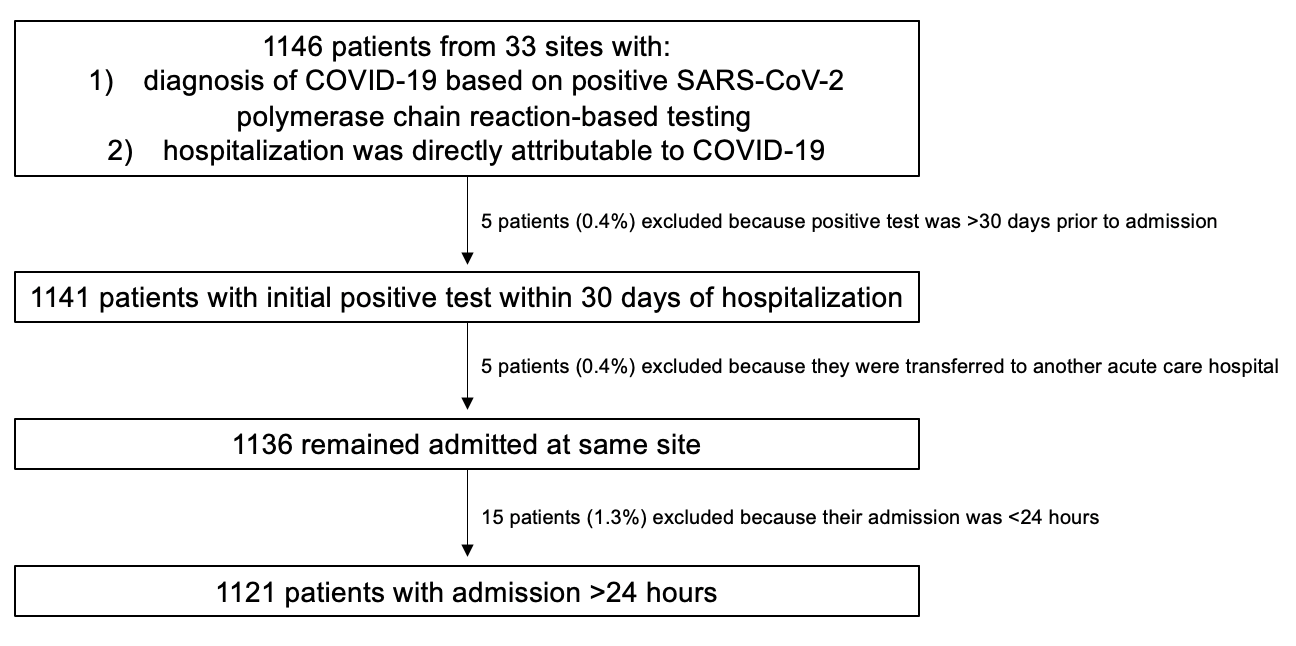


## S2 Table 2: STROBE statement checklist

STROBE Statement—Checklist of items that should be included in reports of ***cohort studies***

|  | Item No | Recommendation | Reported on page |
| --- | --- | --- | --- |
| **Title and abstract** | 1 | (*a*) Indicate the study’s design with a commonly used term in the title or the abstract | 1, 2 |
|  |  | (*b*) Provide in the abstract an informative and balanced summary of what was done and what was found | 2 |
| Introduction | | |  |
| Background/rationale | 2 | Explain the scientific background and rationale for the investigation being reported | 3 |
| Objectives | 3 | State specific objectives, including any prespecified hypotheses | 3 |
| Methods | | |  |
| Study design | 4 | Present key elements of study design early in the paper | 3-6 |
| Setting | 5 | Describe the setting, locations, and relevant dates, including periods of recruitment, exposure, follow-up, and data collection | 3-4 |
| Participants | 6 | (*a*) Give the eligibility criteria, and the sources and methods of selection of participants. Describe methods of follow-up | 3-4 |
|  |  | (*b*) For matched studies, give matching criteria and number of exposed and unexposed | N/A |
| Variables | 7 | Clearly define all outcomes, exposures, predictors, potential confounders, and effect modifiers. Give diagnostic criteria, if applicable | 4-5 |
| Data sources/ measurement | 8* | For each variable of interest, give sources of data and details of methods of assessment (measurement). Describe comparability of assessment methods if there is more than one group | 4-5 |
| Bias | 9 | Describe any efforts to address potential sources of bias | 5-6 |
| Study size | 10 | Explain how the study size was arrived at | 3, Supplement Figure |
| Quantitative variables | 11 | Explain how quantitative variables were handled in the analyses. If applicable, describe which groupings were chosen and why | 5-6 |
| Statistical methods | 12 | (*a*) Describe all statistical methods, including those used to control for confounding | 5-6 |
|  |  | (*b*) Describe any methods used to examine subgroups and interactions | 5-6 |
|  |  | (*c*) Explain how missing data were addressed | 5-6 |
|  |  | (*d*) If applicable, explain how loss to follow-up was addressed | 5-6 |
|  |  | (*e*) Describe any sensitivity analyses | 5-6, Supplement Table 3 |
| Results | | |  |
| Participants | 13* | (a) Report numbers of individuals at each stage of study—eg numbers potentially eligible, examined for eligibility, confirmed eligible, included in the study, completing follow-up, and analysed | 6, Supplement Figure 1 |
|  |  | (b) Give reasons for non-participation at each stage | 3, Supplement Figure 1 |
|  |  | (c) Consider use of a flow diagram | Supplement Figure 1 |
| Descriptive data | 14* | (a) Give characteristics of study participants (eg demographic, clinical, social) and information on exposures and potential confounders | 6-7 |
|  |  | (b) Indicate number of participants with missing data for each variable of interest | N/A |
|  |  | (c) Summarise follow-up time (eg, average and total amount) | 6 |
| Outcome data | 15* | Report numbers of outcome events or summary measures over time | 7-8 |
| Main results | 16 | (*a*) Give unadjusted estimates and, if applicable, confounder-adjusted estimates and their precision (eg, 95% confidence interval). Make clear which confounders were adjusted for and why they were included | 8-9, Figure 1 |
|  |  | (*b*) Report category boundaries when continuous variables were categorized | 4 |
|  |  | (*c*) If relevant, consider translating estimates of relative risk into absolute risk for a meaningful time period | N/A |
| Other analyses | 17 | Report other analyses done—eg analyses of subgroups and interactions, and sensitivity analyses | 8, Supplement Table 3-4 |
| Discussion | | |  |
| Key results | 18 | Summarise key results with reference to study objectives | 10-11 |
| Limitations | 19 | Discuss limitations of the study, taking into account sources of potential bias or imprecision. Discuss both direction and magnitude of any potential bias | 11-12 |
| Interpretation | 20 | Give a cautious overall interpretation of results considering objectives, limitations, multiplicity of analyses, results from similar studies, and other relevant evidence | 10-12 |
| Generalisability | 21 | Discuss the generalisability (external validity) of the study results | 11-12 |
| Other information | | |  |
| Funding | 22 | Give the source of funding and the role of the funders for the present study and, if applicable, for the original study on which the present article is based | 1 |

## S3 Table 3: Adjusted Odds Ratios for total VTE, VTE >48H after admission and VTE <48H after admission

| **Thrombosis event** | **Events/patients, n/1121 (%)** | **Prophylaxis dose (Adjusted OR [95% CI])** | | | |
| --- | --- | --- | --- | --- | --- |
|  |  | **None** | | **Intensified** | **Therapeutic** |
| Total VTE events  PE  DVT  Both  VTE >48H after admission  PE  DVT  Both | 98 (8.7)  47 (4.2)  51 (4.6)  9 (0.8)  62 (5.5)  23 (2.1)  39 (3.5)  5 (0.44) | 1.2 (0.51-2.9)  2.2 (0.84-5.5)  0.90 (0.30-2.8)  --  0.67 (0.21-2.1)  1.0 (0.20-5.1)  0.79 (0.18-3.4)  -- | 0.93 (0.07-12)  empty  1.3 (0.08-21)  --  1.0 (0.06-17)  empty  1.3 (0.06-28)  -- | | 5.2 (2.1-13)  8.7 (4.5-17)  3 (0.79-12)  --  3.0 (0.89-10)  4.4 (2.0-9.5)  2.6 (0.45-15)  -- |
| VTE <48H after admission  PE  DVT  Both | 36 (3.2)  24 (2.1)  12 (1.1)  4 (0.36) | 3.4 (1.1-11)  4.5 (1.2-17)  1.7 (0.32-9.0)  -- | empty  empty  empty  -- | | 14 (5.3-35)  18 (6.4-53)  6.4 (1.2-33)  -- |

**Legend:** Reference category=standard prophylaxis; abbreviations: OR=odds ratio, VTE= venous thromboembolism, CI=confidence interval; adjusted for: chronic lung disease, cardiovascular disease, immunocompromise, diabetes mellitus, end-stage renal disease on dialysis and cancer chronic lung disease, cardiovascular disease, immunocompromise, diabetes mellitus, end-stage renal disease on dialysis and cancer

## S4 Table 4: Prophylaxis dose subgroup analyses

| **Subgroup** | **Prophylaxis dose (Adjusted OR [95% CI])** | | | |
| --- | --- | --- | --- | --- |
|  | **None** | **Intensified** | | **Therapeutic** |
| ICU admission (n=1121) |  | |  |  |
| Yes (n=383) | 1.3 (0.39-4.4) | | 0.56 (0.03-9.2) | 2.6 (0.89-7.4) |
| No (n=738) | 3.1 (0.75-13) | | empty | 16 (5.1-52) |
| Intubated (n=1121)  Yes (n=232)  No (n=889)  Baseline D-dimer (n=815) | 2.0 (0.38-11)  2.0 (0.75-5.2) | | 1.3 (0.06-31)  empty | 2.0 (0.62-6.5)  10 (4.6-22) |
| < 6x ULN (n=700) | 0.99 (0.29-3.4) | | 0.82 (0.05-13) | 3.7 (1.1-12) |
| ≥ 6x ULN (n=115) | 2.4 (0.74-8) | | empty | 12 (5.3-29) |
| Peak D-dimer (n=803) |  | |  |  |
| < 6x ULN (n=604) | 1.4 (0.36-5.7) | | empty | 2.7 (0.58-13) |
| ≥ 6x ULN (n=199) | 1.1 (0.41-2.7) | | 2.4 (0.19-32) | 7.0 (3.6-14) |

**Legend:** Reference category=standard prophylaxis; abbreviations: ULN=upper limit of normal, OR=odds ratio, VTE= venous thromboembolism, CI=confidence interval; adjusted for: chronic lung disease, cardiovascular disease, immunocompromise, diabetes mellitus, end-stage renal disease on dialysis and cancer chronic lung disease, cardiovascular disease, immunocompromise, diabetes mellitus, end-stage renal disease on dialysis and cancer

## S5 Table 5: Patient characteristics of subgroup of 383 patients admitted to the intensive care unit with COVID-19 at 33 US academic medical centers

| **Characteristics** | **ICU patients** |
| --- | --- |
|  | **n=383** |
| Age, mean; SD | 60; 16 |
| Female sex, n (%) | 159 (42) |
| Race ethnicity |  |
| Asian, n (%) | 24 (6.3) |
| Black/African-American, n (%) | 111 (29) |
| Hispanic, n (%) | 108 (28) |
| White, n (%) | 113 (30) |
| Other, n (%) | 27 (7.1) |
| **Anticoagulant agent prior to hospitalization** |  |
| Any anticoagulant, n (%) | 35 (9.1) |
| Warfarin, n (%) | 10 (2.6) |
| DOAC, n (%) | 23 (6.0) |
| LMWH, n (%) | 2 (0.52) |
| **Antiplatelet agent prior to hospitalization** |  |
| Any antiplatelet, n (%) | 98 (26) |
| Aspirin, n (%) | 83 (22) |
| Clopidogrel, n (%) | 11 (2.9) |
| Prasugrel, n (%) | 1 (0.26) |
| Ticagrelor, n (%) | 3 (0.78) |
| **Risk factors** |  |
| Active cancer, n (%) | 20 (5.2) |
| Diabetes, n (%) | 159 (42) |
| Prior VTE, n (%) | 17 (4.4) |
| Prior thrombophilia, n (%) | 3 (0.78) |
| Recent (<30d) trauma or surgery, n (%) | 16 (4.2) |
| ESRD on HD, n (%) | 16 (4.2) |
| Pre-existing lung disease, n (%) | 56 (15) |
| Pre-existing immunosuppression, n (%) | 40 (10) |
| **Risk factors** |  |
|  |  |
| Pre-existing heart failure, n (%) | 49 (13) |
| Pre-existing arterial vascular disease, n (%) | 58 (15) |
| Pre-existing atrial fibrillation, n (%) | 38 (10) |
| Ongoing prothrombotic hormone use, n (%) | 2 (0.52) |
| BMI >/=30, n (%) | 208 (54) |
| **Illness severity** |  |
| Days from symptoms to admission, median (IQR) | 6 (3,9) |
| Intubated, n (%) | 224 (59) |
| Oxygen without intubation, n (%) | 136 (85) |
| ICU LOS, median (IQR) | 7 (3,14) |
| Peak D-dimer, median (IQR) | 3677 (1436,9695) |
| Peak Creatinine, median (IQR) | 1.3 (0.9, 2.9) |
| Padua score, median (IQR) | 6 (5,6) |
| Death during hospitalization, n (%) | 100 (26) |

**Table 1 Legend:** Abbreviations- SD=standard deviation, VTE=venous thromboembolism, d=days, BMI=body mass index, IQR=interquartile range, ICU=intensive care unit, LOS=length of stay

## S6 Table 6: Venous and arterial thromboembolism and bleeding events in subgroup of 383 patients admitted to the intensive care unit with COVID-19 at 33 US academic medical centers

| **Inpatient thrombosis** | **Total events, n/383 (%)** |
| --- | --- |
| Pulmonary embolism, n (%) | 29 (7.6) |
| Peri-PE hypotension, n (%) | 9 (2.3) |
| Right heart strain, n (%) | 11 (2.9) |
| Segmental only, n (%) | 8 (2.1) |
| Days from admission to PE, median (IQR) | 6 (1,14) |
| Deep vein thrombosis, n (%) | 45 (12) |
| Upper extremity, n (%) | 18 (4.7) |
| Lower extremity, n (%) | 27 (7.0) |
| Other, n (%) | 1 (0.26) |
| CVC-associated, n (%) | 10 (2.6) |
| Days from admission to DVT, median (IQR) | 8 (3,18) |
| Arterial thrombosis, n (%) | 17 (4.4) |
| CVA | 10 (2.6) |
| MI | 4 (1.0) |
| Systemic arterial embolism | 3 (0.78) |
| Days from admission to ATE, median (IQR) | 1 (0,10) |
| **Post-discharge thrombosis** |  |
| Post-discharge PE, n (%) | 0 (0) |
| Post-discharge DVT, n (%) | 0 (0) |
| Post-discharge ATE, n (%) | 0 (0) |
| **Inpatient bleeding** |  |
| Major bleed, n (%) | 35 (4.1) |
| MB associated with therapeutic AC, n (%) | 8 (23) |
| CRNMB, n (%) | 30 (4.1) |
| CRNMB with therapeutic AC, n (%) | 13 (43) |
| Number of pRBC units transfused, median (IQR) | 3 (1,7) |
| **Post-discharge bleeding** |  |
| Post-discharge MB, n (%) | 1 (0.26) |
| Post-discharge MB with therapeutic AC, n (%)  Days from discharge to MB, median (IQR) | 1 (100)  27 (27,27) |

**Table 3 Legend:** Abbreviations- ICU=intensive care unit, H=hours, PE=pulmonary embolism, IQR=interquartile range, CVC=central venous catheter, DVT=deep vein thrombosis, CVA=cerebrovascular accident, MI=myocardial infarction, ATE=arterial thromboembolism, MB=major bleed, AC=anticoagulation, CRNMB=clinically-relevant non-major bleed, pRBC=packed red blood cells
